# Thermal Inactivation of Multiple Veterinary-Relevant Viruses: Effects of Environmental Conditions, Surface Type, and Organic Matrix

**DOI:** 10.3390/pathogens14121243

**Published:** 2025-12-05

**Authors:** Isac Junior Roman, Ingryd Merchioratto, Renata Nobre da Fonseca, Mayara Fernanda Maggioli, Fernando Vicosa Bauermann

**Affiliations:** 1 Department of Veterinary Pathobiology, College of Veterinary Medicine, Oklahoma State University (OSU), Stillwater, OK 74078, USA; 2 Laboratório de Doenças Parasitárias (LADOPAR), Departamento de Medicina Veterinária Preventiva (DMVP), Centro de Ciências Rurais (CCR), Universidade Federal de Santa Maria (UFSM), Santa Maria 97105-900, RS, Brazil; 3 Lab Plataforma de Investigación en Salud Animal, Instituto Nacional de Investigación Agropecuaria (INIA), Colonia 70006, Uruguay

**Keywords:** biosecurity, disinfection, thermo-assisted decontamination, virus

## Abstract

Heat is widely used to decontaminate livestock environments, yet performance varies with virus, surface, moisture, and organic load. We evaluated the effects of temperature (50, 60, 70 °C) and exposure time on the viability of 10 veterinary-relevant viruses (or surrogates) placed on four nonporous surfaces (plastic, rubber, aluminum, stainless steel) under dry or wet conditions, and in organic matrices (blood, wheat straw, complete feed). Infectivity was quantified by TCID_50_ using independent duplicate experiments with duplicate titrations. Moist heat consistently outperformed dry heat: at 60–70 °C, all enveloped viruses, and most non-enveloped viruses were inactivated on surfaces within 5 min, while porcine parvovirus (PPV) remained the outlier, requiring ≥60 min. In contrast, dry heat allowed several viruses to persist for 24 h at 70 °C, underscoring that temperature alone is an unreliable predictor of rapid decontamination in the absence of humidity. Organic matrices modulated outcomes in a substrate- and virus-dependent manner, with some combinations accelerating inactivation and others prolonging survival to ≥180 min at ≥60 °C. These findings support matrix-aware, heat-assisted protocols for facilities and transport (e.g., 70 °C for ≥10 min under high humidity for most enveloped viruses), while recognizing exceptions such as PPV. The data provide actionable parameters to optimize thermo-assisted decontamination in veterinary systems.

## 1. Introduction

The livestock sector supports global supplies of animal protein yet remains vulnerable to infectious diseases. Viral outbreaks can propagate rapidly across integrated production networks, resulting in reproductive losses, increased mortality, and decreased productivity. The resulting effect cascades through supply chains, with substantial economic impacts and material risks to food security [1,2]. Among livestock species, swine production may be particularly susceptible, as the intensive, high-density, and globally connected nature of pig farming increases the likelihood and significance of viral disease impacts [3,4,5]. Cattle operations likewise face substantial risk, given the scale of international trade and concentration in feedlots, which facilitate the spread of endemic and emerging viral pathogens [6,7].

Once introduced, viruses excreted by infected animals can persist in the environment, with their viability influenced by both viral properties and environmental conditions [8,9,10]. Although commercial cattle and, especially, swine production systems typically maintain strict control over entry and exit from production units [11,12,13], there are still potential mechanisms that may serve as links for disease transmission [14,15]. 

Surfaces made of materials such as plastic, steel, aluminum, and rubber are commonly found in facilities, including pens, feeders, personal protective equipment (PPE), storage, and trucks [16,17]. If not properly decontaminated, these materials can become sources of infection. This is particularly relevant for transportation, which may act as an indirect dissemination route among the different production units [14,18,19]. Ineffective decontamination of transport vehicles played a major role in the spread of both porcine reproductive and respiratory syndrome virus (PRRSV) and porcine epidemic diarrhea virus (PEDV) outbreaks [20,21].

Among methods to inactivate viruses, heat inactivation has long been used as a primary tool for decontamination and sterilization in various settings, including installations, at multiple stages of the food processing chain, and in the biomedical field [22,23]. Heat inactivation of viruses is believed to occur through the denaturation of protein and other molecular secondary structures, impairing molecular function [24]. Typically, the higher the treatment temperature, the less exposure time is needed to inactivate viruses [19,25]. The specific characteristics of each virus and the material (matrix) in which they are contained are also critical factors influencing time to inactivation. Likewise, the properties of the material undergoing treatment may limit the use of high temperatures, extending the time needed for disinfection [26,27,28].

The usefulness of thermal inactivation of viruses led to its application for swine trailer decontamination [19,20,21,29]. Thermo-assisted drying and decontamination (TADD) protocols typically operate at 63–70 °C for up to 30 min. The method was successfully employed to inactivate PRRSV and PEDV in trailers under field conditions [29,30]. Similar treatment protocols were used to test the thermal treatment of other pathogens under experimental conditions, including swine influenza virus (SIV) and rotavirus [19].

The use of thermal treatment to inactivate viruses may be a cost-effective option in various parts of the livestock-producing chain, helping to curb the spread of pathogens and during outbreaks of high-impact animal diseases or newly emerging pathogens. Therefore, it is crucial to understand the efficacy of heat treatment against various viruses to implement rational and effective measures and establish protocols that enhance preparedness and response readiness. However, there is a lack of comparative data on the thermal inactivation of an extensive range of viruses, including both enveloped and non-enveloped viruses, under the same conditions. These are vital for developing heat-based decontamination strategies.

Given this gap in the existing evidence base, the present study evaluates 10 veterinary-relevant viruses (Table 1) or family-matched surrogates under combinations of temperature, moisture, surface, and organic matrices. Our objective was to investigate the inactivation dynamics across these conditions and to translate the resulting patterns into actionable guidance for livestock facilities, transportation, sanitation, and outbreak response.

## 2. Materials and Methods

### 2.1. Viruses and Cells

The study evaluated the efficacy of heat treatment under various simulated conditions for 10 viruses listed in Table 1. The group of non-enveloped positive-sense RNA viruses included feline calicivirus (FCV), used as a surrogate for vesicular exanthema of swine virus (VESV) of the *Caliciviridae* family; senecavirus A (SVA), a surrogate for foot-and-mouth disease virus (FMDV) from the *Picornaviridae* family; and porcine sapelovirus (PSV), which served as a surrogate for swine vesicular disease virus (SVDV), also belonging to the *Picornaviridae* family. Among the enveloped positive-sense RNA viruses, the study analyzed PEDV from the *Coronaviridae* family; bovine viral diarrhea virus (BVDV), which acted as a substitute for classical swine fever virus (CSFV) from the *Flaviviridae* family; and PRRSV, representing the *Arteriviridae* family. The enveloped negative-sense RNA viruses investigated included SIV of the *Orthomyxoviridae* family and canine distemper virus (CDV), which was used as a surrogate for Nipah virus (NiV) of the *Paramyxoviridae* family. The non-enveloped DNA virus included was the porcine parvovirus (PPV), a member of the *Parvoviridae* family. Finally, the group of enveloped double-stranded DNA viruses comprised bovine herpesvirus type 1 (BoHV-1), which served as a substitute for pseudorabies virus (PRV) from the *Herpesviridae* family.

Cell cultures were maintained in minimal essential medium (MEM; Corning, Mediatech, Inc., Manassas, VA, USA) supplemented with 10% fetal bovine serum (FBS; Seradigma^®^, VWR International, LLC, Radnor, PA, USA), 2 mM L-glutamine (Corning), 1% Antimycotic–Antibiotic 100× (Gibco™, Grand Island, NY, USA), and 50 µg/mL gentamicin (Corning). PEDV and SIV were propagated in FBS-free medium supplemented with 2 µg/mL TPCK-trypsin (L-1-tosylamido-2-phenylethyl chloromethyl ketone; Sigma-Aldrich^®^, St. Louis, MO, USA), while PRRSV was amplified in medium containing 5% horse serum (Gibco™). All cultures were incubated at 37 °C in a humidified atmosphere with 5% CO_2_.

Virus stocks were titrated using the Reed and Muench method, and titers were expressed as the median tissue culture infectious dose per milliliter (TCID_50_/mL). Details of the cell lines employed and the viral titers obtained are provided in Table 1.

### 2.2. Study Design

We evaluated thermal inactivation of ten veterinary-relevant viruses (enveloped and non-enveloped) under conditions chosen to reflect decontamination scenarios in livestock production. Primary experiments were performed at 70 °C using extended exposures to characterize viability over time on nonporous surfaces and in organic substrates. Based on these results, a subset of informative time points was then tested at 50 °C and 60 °C. Since each virus–matrix pair exhibits unique inactivation kinetics, time points were tailored for each combination rather than being fixed across the study.

Matrices comprised common nonporous surfaces (plastic, rubber, aluminum, and stainless steel) under dry and wet conditions, as well as organic substrates typical of livestock environments (whole blood, bedding, and complete feeds). After each heat exposure, the samples were immediately chilled and subjected to virus titration. All conditions were evaluated in two biological replicates with duplicate titrations, alongside room-temperature (~20–22 °C) controls processed in parallel. Experimental protocols and matrix-specific handling are detailed in Section 2.3, Section 2.4 and Section 2.5.

### 2.3. Virus Inactivation on Surfaces Under Dry and Wet Conditions

Flat surfaces of plastic, rubber, aluminum, and stainless steel (2.5 cm^2^ each) were thoroughly cleaned with distilled water to remove chemical or organic residues, sterilized in an autoclave, and subsequently inoculated with 100 µL of undiluted virus. For dry conditions, the inoculum was allowed to air-dry for approximately 50 min at room temperature inside a Class A2 biosafety cabinet before being subjected to thermal treatment protocols for up to 1440 min. Studies were conducted under controlled environmental conditions, with relative humidity ranging from 25% to 35%. For wet conditions, the inoculated surfaces were immediately transferred to a humidity chamber prepared with absorbent paper soaked in preheated distilled water, ensuring the internal environment matched the incubator temperature. A wireless thermometer probe was placed inside the chamber to monitor temperature throughout the treatment (for up to 180 min). To avoid pressure buildup from evaporating water, the chamber was not completely sealed. Following the designated incubation time under either condition, viral material from each surface was recovered by adding 1 mL of MEM.

### 2.4. Virus Inactivation in Organic Matter

To evaluate the influence of organic matter on viral inactivation by thermal treatment, viruses were mixed with organic substrates at the following ratios: (A) 50 µL of virus with 450 µL of whole blood collected in Alsever’s purchased from Pel-Freez Biologicals, Rogers, AR. To minimize interference from pre-existing, species-specific antibodies, we used heterologous blood matrices: bovine blood for porcine viruses and swine blood for non-porcine viruses. (B) 25 µL of virus with 225 mg of bedding (wheat straw), and (C) 50 µL of virus with 450 mg of feed (starter or finisher). Because swine feed composition may affect viral stability, two complete feed formulations were used: starter feed containing powdered milk (21% protein, ~1.5% lysine) and finisher feed lacking powdered milk (≤16% protein, ≤0.8% lysine). For reference, the starter feed provided also contained 3.9% crude fat, and no more than 5% crude fiber, with calcium ranging from 0.40–0.90%, phosphorus ≥ 0.45%, salt 1.00–1.50%, selenium ≥ 0.30 ppm, and zinc ≥ 3000 ppm. The finisher feed also contained 2.50% crude fat, and ≤14% crude fiber, with calcium ≤ 0.75%, phosphorus ≥ 0.50%, salt 0.50–1.00%, sodium 0.20–0.70%, selenium ≥ 0.30 ppm, and zinc ≥ 150 ppm.

Mixtures were incubated for up to 180 min in 50 mL conical tubes (for feed and wheat straw) or 1.5 mL microtubes (for blood). After treatment, feed and wheat straw samples were recovered using 2 mL of MEM, and blood samples were recovered using 1 mL of MEM. Tubes were vigorously shaken and centrifuged at 10,000× *g* for 5 min. The supernatant was collected, placed on ice, and immediately subjected to viral titration.

### 2.5. Virus Titration

Viral titrations were performed in duplicate for each independent biological replicate. Samples with titers below the assay threshold (10^1.9^ TCID_50_/mL) were denominated inactivated. Figures were prepared using GraphPad Prism (version 9.2.0).

Cytopathic effects (CPE) were evaluated under an inverted light microscope, except for PRRSV, for which an additional indirect immunofluorescence assay (IFA) was conducted to confirm the results. For the IFA, the culture medium was discarded, and 100 µL/well of 3–4% formaldehyde inPBS was added for fixation (30 min, room temperature), followed by three washes with PBS. Cell membranes were permeabilized with 100 µL/well of 0.2% Triton X-100 in PBS (30 min, room temperature), followed by three additional washes. The primary antibody (PRRSV-NA Mab, VMRD, Pullman, WA, USA) was diluted 1:300 in 1% BSA–PBS, and 50 µL was added per well (60 min, 37 °C), followed by three washes. The secondary antibody (Goat anti-mouse IgG H+L, Alexa Fluor™ 488 A11001, Invitrogen, Thermo Fisher Scientific, Waltham, MA, USA) was diluted 1:300 in 1% BSA–PBS, and 50 µL was added per well (60 min, 37 °C), followed by three washes. Finally, 100 µL of PBS was added per well, and virus-induced cytopathic effects (CPE) were assessed in cell monolayers by fluorescent antibody (FA) staining and imaged on an inverted epifluorescence microscope.

## 3. Results and Discussion

To evaluate the thermal stability of veterinary-relevant viruses under operationally realistic conditions, we exposed virus-inoculated materials to controlled temperatures (50 °C, 60 °C, 70 °C) across three environmental conditions: dry surfaces, wet conditions, and organic matrices. The experimental design captured diverse settings encountered in livestock facilities, transportation equipment, and contaminated feed or bedding.

**Figure 1:**
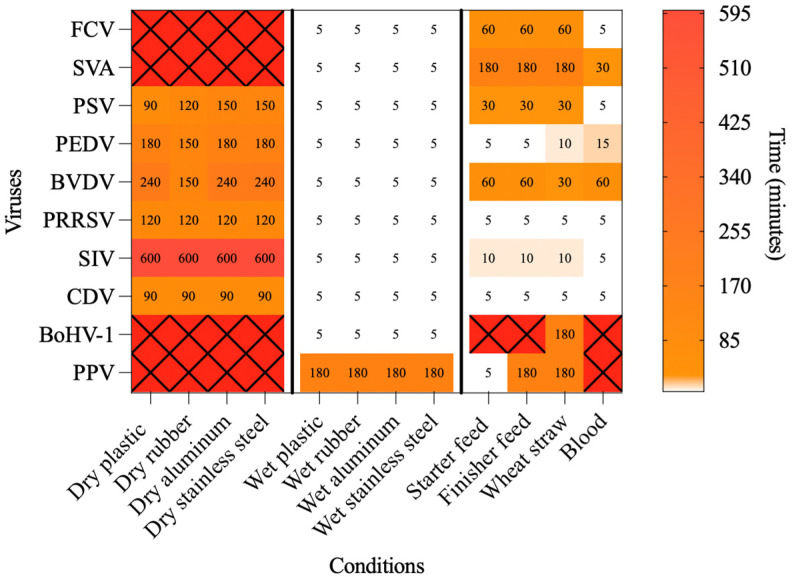
Heatmap showing the time required to inactivate each of the tested viruses under the dry, wet, and organic conditions at 70 °C. White cells indicate virus–matrix combinations in which inactivation occurred within less than 5 min. Red cells marked with an “X” indicate virus–matrix combinations in which no inactivation was observed up to the maximum time tested (1440 min for dry conditions and 180 min for wet and organic conditions).

**Figure 2:**
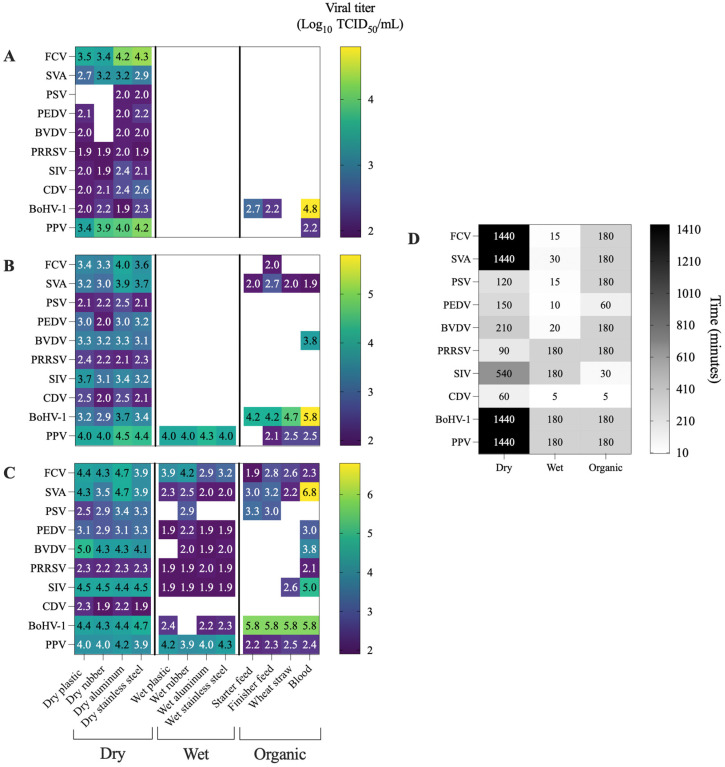
Heatmaps displaying the viral titers obtained for each virus under different combinations of temperature and matrix conditions. Heatmap compares viral titers measured at the same time point across the three temperatures tested. The selected time point corresponds to the latest sampling point at which viral detection remained above the assay threshold (1.9 log_10_ TCID_50_/mL) for at least one temperature–surface combination. White cells indicate viral inactivation, defined as titers below the detection limit (1.9 log_10_ TCID_50_/mL). (**A**) viral titers at 70 °C; (**B**) viral titers at 60 °C; (**C**) viral titers at 50 °C; (**D**) time point selected for cross-temperature comparisons.

Figure 1 and Figure 2 provide the viral inactivation dynamics across all tested viruses, temperatures, and surface types. Since viability patterns varied widely depending on the virus and tested conditions, the results are presented by grouping viruses according to their envelope and genome. This organizational approach distinguishes between enveloped and non-enveloped RNA and DNA viruses, allowing for comparison of stability patterns. The viruses used as surrogate models in this study are supported by previous literature [31,32,33,34,35,36,37]. Moreover, surrogate viruses employed in the study are also pathogens with significant veterinary importance.

### 3.1. Non-Enveloped +ssRNA Viruses (FCV, SVA, PSV)

**Figure 3:**
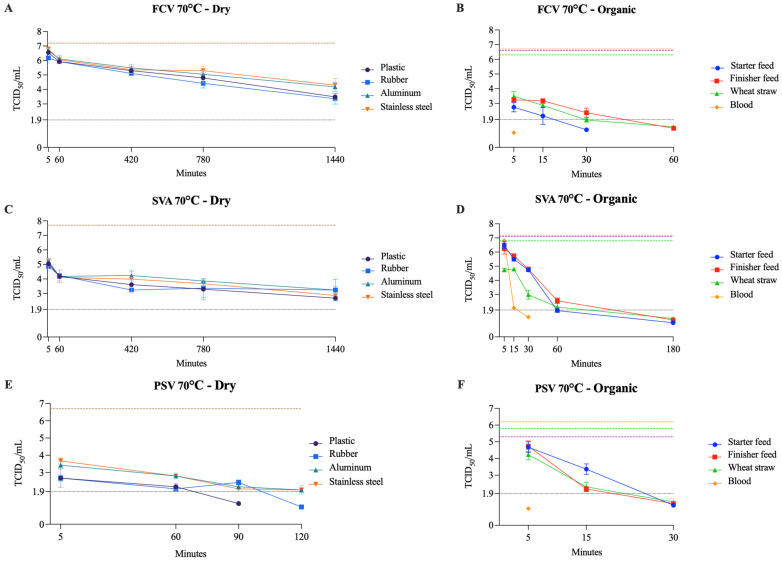
Viability of FCV (**A**,**B**), SVA (**C**,**D**), and PSV (**E**,**F**) at 70 °C on dry surfaces and organic materials, respectively. The black dotted line represents the limit of viral detection (<1.9 log_10_ TCID_50_/mL). In the dry surface graphs (**A**,**C**,**E**), the brown dashed line represents the initial viral titer (log_10_ TCID_50_/mL) with the included dilution factor for each matrix. In the organic material graphs (**B**,**D**,**F**), the orange, purple, and green dashed lines indicate the initial viral titer (log_10_ TCID_50_/mL) for blood, feed (finisher and starter), and wheat straw, respectively.

Dry conditions within this group consistently extended the viability of FCV and SVA, which remained infectious for over 24 h at 50–70 °C on all surfaces. In contrast, PSV was inactivated between 90 and 150 min at 70 °C across all surfaces. Under wet conditions, inactivation occurred rapidly, with all three viruses inactivated within 5 min or less at 70 °C and 60 °C on all surfaces. In organic matrices, these viruses were inactivated within 5 to 180 min at 70 °C, but at 60 °C, SVA continued to be infectious until the final testing point across all surfaces. Overall, PSV proved more sensitive to temperature–surface combinations, whereas SVA was more resistant. Humidity also appears to significantly influence inactivation, while blood among organic matrices apparently accelerates the process compared to solid organic matrices. Table 2 presents details on the three different temperatures tested, along with the time points and viral titers for FCV, SVA, and PSV. The viability of FCV, SVA, and PSV at 70 °C on dry surfaces and organic matrices is shown in Figure 3.

These findings align with previous studies that demonstrated that FCV remained viable for up to 7 days at room temperature on a stainless steel surface [38]. In another study, FCV was detected at a significantly higher frequency on solid, dry surfaces (e.g., food bowls, ventilation filters) than in water and litter trays [39]. Moreover, it has been shown that a relative humidity of 30% provides a better environment for FCV survival than 70% [40]. These results support our findings that dry surfaces are more permissive for FCV surveillance than wet surfaces. In addition, FCV served as a surrogate for VESV, for which data on heat inactivation are largely unavailable. To date, only one study with a similar experimental design has reported a 2–3 log_10_ reduction after exposure to 50 °C for 1 h in water suspension [41].

SVA showed strong resistance across all dry surfaces examined. However, this resistance was strongly reduced under wet conditions. It was shown that lower temperatures are less effective for inactivating SVA in swine feed ingredients (dried distillers’ grains with soluble, soybean meal, and lysine). Among 4, 15, and 30 °C, the lowest temperature resulted in the longest estimated time for the virus to decay to 0.01% [42]. Similarly, SVA remained viable for up to 90 days in spleen and bone marrow tissues at 5.5 °C, which was significantly reduced (up to 45 days) when incubating at 29.9 °C [33]. These results were similar to ours for organic matrices (feed and blood), where titer decayed faster at 70 °C than at 50–60 °C. For FMDV, studies have shown that the effective inactivation temperature is approximately 60 °C in a PBS suspension [43], and on dry glass and plastic surfaces for up to 1 day at 70 °C and up to 7 days at room temperature [44]. Our results with SVA were slightly different; on a dry plastic surface, complete viral inactivation was not achieved at 70 °C after 24 h, although there was a significant reduction in viral titer, from 10^7.7^ to 10^2.6^ TCID_50_/mL.

PSV was shown to be effectively inactivated at 54 °C within 6 min, resulting in a complete reduction in the viral titer [45]. Similar results were found by [46], demonstrating that PSV was inactivated by heating at 60 °C for 10 min or 65 °C for 5 min, with no CPE detected afterward. These results are similar to those observed when treating PSV with wet heat at 50 °C in aluminum and at 60–70 °C on all surfaces tested. However, since the cited authors did not specify the surface, comparisons should be taken with caution. Interestingly, PSV was inactivated within 5 min at 70 °C when the organic matrix used was blood. Although there is a gap in the literature regarding SVD inactivation, two studies have shown that SVD is inactivated in organic matrices such as pig slurry and plasma when treated at 65 °C for 2 min [47] and spray-drying [48], respectively.

### 3.2. Enveloped +ssRNA Viruses (PEDV, BVDV, PRRSV)

**Figure 4:**
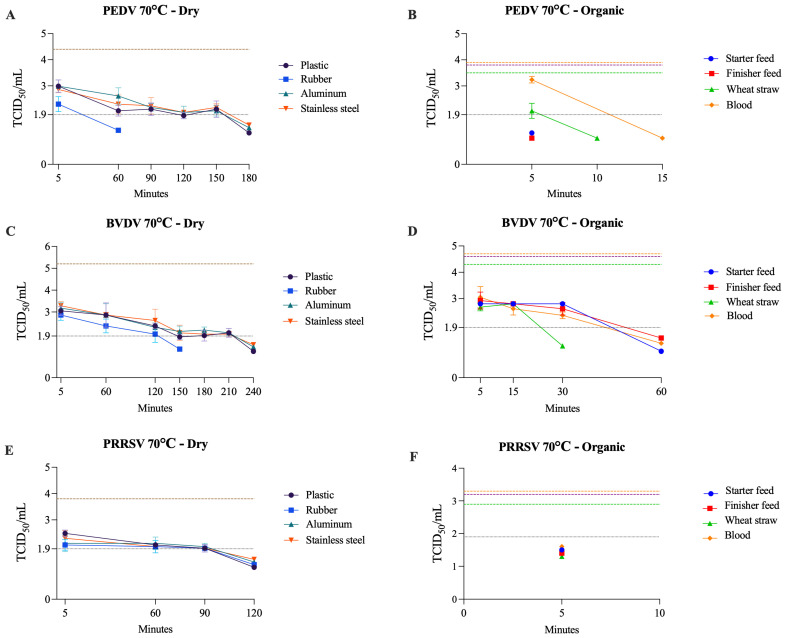
Viability of PEDV (**A**,**B**), BVDV (**C**,**D**), and PRRSV (**E**,**F**) at 70 °C on dry surfaces and organic materials, respectively. The black dotted line represents the limit of viral detection (<1.9 log_10_ TCID_50_/mL). In the dry surface graphs (**A**,**C**,**E**), the brown dashed line represents the initial viral titer (log_10_ TCID_50_/mL). In the organic material graphs (**B**,**D**,**F**), the orange, purple, and green dashed lines indicate the initial viral titer (log_10_ TCID_50_/mL) for blood, feed (finisher and starter), and wheat straw, respectively.

Moist heat was highly effective against all three viruses, with inactivation observed within 5 min at 60–70 °C. Under dry conditions, PEDV, BVDV, and PRRSV persisted for up to 240 min at 70 °C, highlighting a protective effect. In the organic matrices, the inactivation time ranged from 5 to 60 min at 70 °C (Table 3). Among these three viruses, BVDV appears to be the most resistant to both temperature and surface type, except on wet surfaces. Notably, in blood, it was the only one not inactivated until the last time point at 60 °C. Additionally, in both dry and organic matrices, it required the longest exposure time for inactivation at 70 °C. A similar pattern, observed in non-enveloped +ssRNA viruses, was observed here for wet surfaces, with inactivation within 5 min at 70 °C. Conversely, in this case, the blood did not accelerate the inactivation process compared to other solid organic matrices. The thermal inactivation profiles of PEDV, BVDV, and PRRSV at 70 °C on organic matrices and dry surfaces are shown in Figure 4.

Previous studies showed that PEDV has high thermal resistance in dry feed samples, requiring higher temperatures to be inactivated (~3 log reduction) faster (e.g., 10 min at 145 °C and 25 min at 120 °C) [49]. The same pattern was observed when evaluating PEDV thermal resistance on various feed ingredients. It was found that the maximum log reduction (3.9 log) occurred at 90 °C for 30 min [50]. These findings showed discrepancies with our results, in which PEDV was inactivated within 5 min at 50, 60, and 70 °C across both starter and finisher feed formulations. Methodological differences might account for this discrepancy. Our experiments used complete feed formulation, whereas a previous study assessed individual feed ingredients [50], which may not replicate the heat transfer and moisture characteristics of complete feed matrices. Additionally, variations in feed composition can affect the stability of viral inactivation [51]. In our study, temperature did not significantly affect the reduction in PEDV titer on plastic, stainless steel, or aluminum dry surfaces. Another study reported that PEDV remained viable on all three surfaces for up to 10 days at room temperature [50]. An additional study reported that 15 min at 50 °C or 6 h at 30–40 °C on a dry aluminum surface inactivated PEDV [52].

Our findings indicate that BVDV exhibits greater resistance to heat and organic matrices than both PRRSV and PEDV. A previous study reported that BVDV remained viable at 5.5 °C for up to 75 days in the spleen [33] and at 5 °C for up to 3 weeks in bovine slurry [53]. However, the latter was inactivated within 5 min when the temperature was increased to 50 °C [53]. Meanwhile, CSFV appeared to be more resistant at 5 °C, requiring more than 6 weeks for inactivation [53]. In serum, BVDV showed no decrease in titer for at least 5 days at 22 °C [54] and for 30 min at 56 °C [55]. On a dry stainless steel surface, BVDV was inactivated when incubated at 95 °C for 2 h [56]. These results highlight that BVDV sensitivity is both temperature- and surface-specific—i.e., matrix-specific.

PRRSV required 36 h at 30 °C, 24 h at 40 °C, and 6 h at 50 °C to achieve inactivation on a dry aluminum surface [52]. For dry plastic, rubber, and stainless steel surfaces, a previous study [57] demonstrated that incubation for >30 min and <24 h at room temperature was sufficient to inactivate PRRSV, consistent with our findings. A study using the TADD system demonstrated that washing trailers and exposing them to 71 °C for 30 min completely eliminated detectable PRRSV RNA. Conversely, when trailers were either washed without drying or air-dried without heat application, viral RNA was still detectable in samples collected up to 30 min post-treatment [29]. Moreover, an exponential decline in PRRSV infectivity with increasing temperature has been reported in pig manure, with the virus showing a shorter half-life in manure than in MEM across temperatures from 4 to 80 °C [58]. These results are consistent with our findings and further indicate that PRRSV is sensitive to both elevated temperatures and organic matrices.

### 3.3. Enveloped −ssRNA Viruses (SIV, CDV)

**Figure 5:**
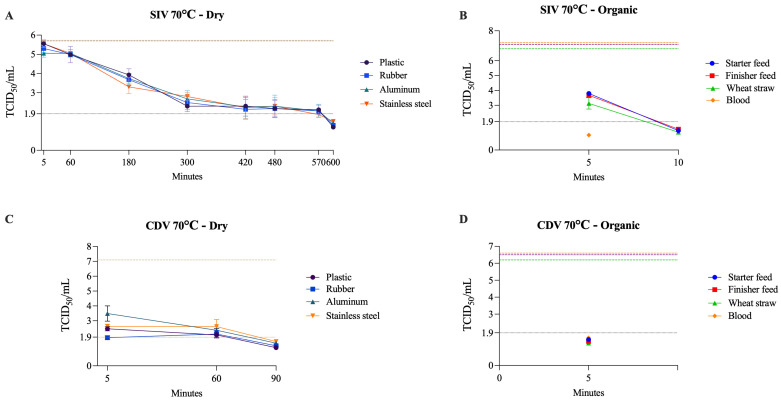
Viability of SIV (**A**,**B**) and CDV (**C**,**D**) at 70 °C on dry surfaces and organic materials, respectively. The black dotted line represents the limit of viral detection (<1.9 log_10_ TCID_50_/mL). In the dry surface graphs (**A**,**C**), the brown dashed line represents the initial viral titer (log_10_ TCID_50_/mL). In the organic material graphs (**B**,**D**), the orange, purple, and green dashed lines indicate the initial viral titer (log_10_ TCID_50_/mL) for blood, feed (finisher and starter), and wheat straw, respectively.

SIV exhibited the most extended dry-state survival among RNA enveloped viruses, remaining infectious for up to 570 min at 70 °C. In contrast, CDV persisted for 60 min under the same conditions. Similar to previous results, moisture markedly increased heat susceptibility for both viruses. CDV was inactivated within 5 min on all organic matrices (50–70 °C), whereas SIV persisted in blood and wheat straw for up to 30 min at 50 °C (Table 4). Overall, SIV showed greater resistance to dry and organic surfaces than CDV. Figure 5 illustrates the viability of SIV and CDV at 70 °C under both dry and organic conditions.

SIV was reported to be inactivated at 50 °C for approximately 30 min in liquid manure [59], and required only 3 min at 55 °C, and less than 1 min at 60 to 80 °C when incubated in MEM [19]. When incubated in distilled water, SIV persisted for up to 250 days at 4 °C. However, at 37 °C, the persistence time decreased to 3.4 days [60]. These results suggest that high temperatures and moisture may be key factors in SIV inactivation, which matches our observations. A similar experiment was made using human H1N1 (Influenza A/Puerto Rico/8/34). Influenza A virus suspensions were dried on stainless steel surfaces and exposed to 55–65 °C at 25–75% relative humidity (RH) for up to 1 h [61]. Results demonstrated that inactivation of influenza virus on stainless steel surfaces increased with higher temperatures and humidity, exceeding 5 log_10_ reduction at 60–65 °C and 50–75% within 30–60 min [61]. In comparison, our results showed that SIV exposed to 60 °C on a wet stainless steel surface experienced a reduction of more than 3.8 log_10_ within 5 min (titer below the assay threshold).

CDV is an important veterinary pathogen and has been used as a potential surrogate for NiV. Based on limited studies, the estimated viability of CDV at 5, 25, 37, and 56 °C was 3–4 days, 10 h, 1 h, and 6 min, respectively [62]. Experimental data on NiV indicate that heating at 56 °C for 60 min, or 60 °C for 30 min in 90% human serum, was sufficient to reduce viral titers below the detection threshold (< 1.39 × 10^2^ TCID_50_/mL) [63], and that 5 min at 85 °C incubation resulted in complete viral inactivation [64]. Similarly, in our study, the CDV titer was reduced to <10^1.9^ TCID_50_/mL within 5 min when incubated with blood at 50–70 °C, which indicates rapid inactivation of CDV in organic matrices.

### 3.4. Enveloped dsDNA Virus (BoHV-1)

**Figure 6:**
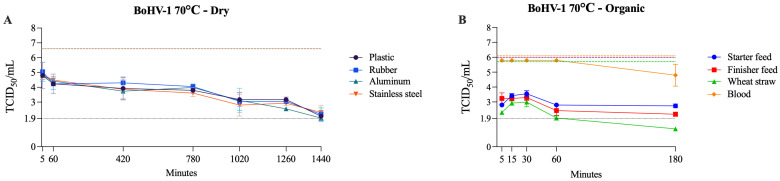
Viability of BoHV-1 at 70 °C on dry surfaces and organic materials. The black dotted line represents the limit of viral detection (<1.9 log_10_ TCID_50_/mL). In the dry surface graph (**A**), the brown dashed line represents the initial viral titer (log_10_ TCID_50_/mL). In the organic material graph (**B**), the orange, purple, and green dashed lines indicate the initial viral titer (log_10_ TCID_50_/mL) for blood, feed (finisher and starter), and wheat straw, respectively.

BoHV-1 showed strong resistance under dry and organic conditions, remaining detectable until the last tested time points (1440 min for dry surfaces and 180 min for organic matrices) at 70 °C. An exception was wheat straw, on which BoHV-1 was inactivated within 180 min at 70 °C. In contrast to RNA viruses, which were generally inactivated within 60 min or less on all organic surfaces, BoHV-1 viability was prolonged. Additionally, unlike the pattern seen with other viruses, BoHV-1 was not inactivated in blood within 180 min at 70 °C. On wet surfaces, its inactivation pattern followed the general trend, with complete inactivation occurring within 5 min at 70 °C and within 180 min or less at 60 °C (Table 5). The viability of BoHV-1 at 70 °C on dry surfaces and organic matrices is shown in Figure 6.

One previous study indicated that BoHV-1 was not inactivated when incubated at 56 °C for 60 min in a water bath [65]. Another study showed that BoHV-1 remained viable for up to 60 days in the spleen at 5.5 °C [33], whereas PRV remained viable for up to 37 days in pork sausage casings at 3 to 16 °C [66]. Moreover, PRV displays notable environmental stability, withstanding diverse temperature and substrate conditions. The virus can remain viable for several weeks in slurry, soil, hay, and straw, with longer persistence observed at lower temperatures [67]. Specifically, PRV survived up to 27 weeks in slurry at 4 °C and 15 weeks at 23 °C, and remained detectable in soil for 5–6 weeks. In food waste, viral inactivation occurred within 24 h at 20–30 °C, but required up to 96 h at 5 °C [67]. Collectively, these observations support the use of BoHV-1 as a surrogate for PRV, given the similar patterns of persistence and resistance to temperature and environmental stressors.

### 3.5. Non-Enveloped DNA Virus (PPV)

**Figure 7:**
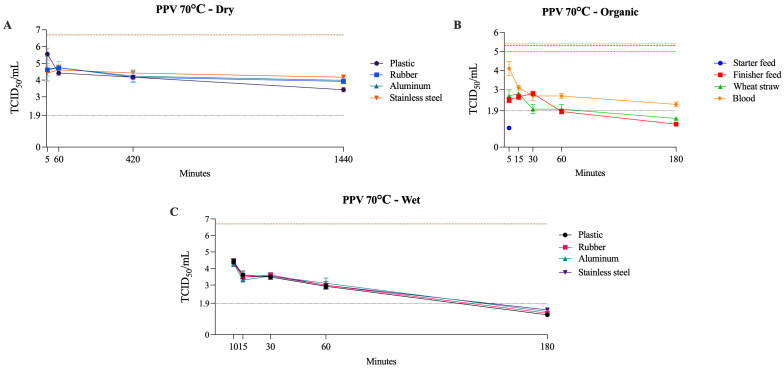
Viability of PPV at 70 °C on dry surfaces, wet surfaces, and organic materials. The black dotted line represents the limit of viral detection (<1.9 log_10_ TCID_50_/mL). In the dry and wet surface graphs (**A**,**C**), the brown dashed line represents the initial viral titer (log_10_ TCID_50_/mL). In the organic material graph (**B**), the orange, purple, and green dashed lines indicate the initial viral titer (log_10_ TCID_50_/mL) for blood, feed (finisher and starter), and wheat straw, respectively.

PPV was the most heat-resistant virus evaluated. Under dry conditions, it remained viable for up to 24 h at 50–70 °C, and under wet conditions, it persisted for up to 180 min at 70 °C with no evidence of inactivation within the same time frame at 50–60 °C. In organic matrices at 70 °C, its behavior varied across materials, as PPV was inactivated within 5 min in starter feed, required up to 180 min in finisher feed and wheat straw, and remained viable in blood at the last tested time point (Table 6). This pattern differed markedly from that observed for the other viruses tested and was most similar to BoHV-1, although PPV demonstrated even greater resistance. Figure 7 presents PPV viability at 70 °C across dry, wet, and organic conditions. 

Reports indicate that PPV was inactivated in a pancreatin suspension after heating at 60 °C for 5 h [68]. In contrast, no inactivation occurred when PPV was incubated in serum albumin suspension at 60 °C for 1 h [69], nor in pig skin, where PPV remained viable throughout the entire tested period (5 h at 62 °C) [70]. This pronounced resistance is consistent with the structural features of the PPV capsid, which is small at approximately 25–28 nm, non-enveloped, and assembled from 60 tightly packed VP1 and VP2 subunits that form a rigid icosahedral shell [71,72]. This compact, highly stable architecture provides substantial protection for the viral genome. It contributes to the virus’s ability to withstand harsh environmental and physical conditions, including low pH, desiccation, and elevated temperatures [73].

## 4. Conclusions

Thermal inactivation is under-characterized for many viruses, particularly veterinary pathogens, and heterogeneous experimental approaches have hindered meaningful comparisons across studies. To address this, we evaluated multiple veterinary viruses in parallel under harmonized, side-by-side conditions spanning temperatures, humidity states, and representative matrices. These aligned measurements complement prior reports and provide directly comparable benchmarks for practical application.

Overall, dry surfaces showed the highest resistance to viral inactivation across all temperatures (50–70 °C) tested. Meanwhile, it was observed that moisture significantly reduces the inactivation time on these surfaces, such as reducing it from 600 min to 5 min for SIV on all surfaces at 70 °C. The organic matrices provided the greatest viability, with outcomes strongly dependent on the specific type of matrix used.

Studies consistently show that virus viability in organic matrices is highly variable and strongly influenced by the physicochemical characteristics of each material. Organic substrates such as feed components, serum-rich fluids, and manure often prolong viral survival by providing protective protein and lipid shielding, altering water activity, and creating microenvironments that buffer thermal or chemical stress. However, this protective effect is not uniform, as intrinsic properties of each matrix can either stabilize virions or accelerate their decay [26,74,75]. These findings underscore that viral persistence in organic materials cannot be generalized and instead reflects complex interactions between the virus and the specific matrix in which it is embedded.

Across the non-enveloped RNA viruses tested (FCV, SVA, and PSV), FCV and SVA remained viable on dry surfaces for up to 24 h at 70 °C, whereas PSV required up to 150 min for inactivation under the same conditions. In contrast, all three viruses were inactivated within 5 min at 70 °C under wet conditions. In the organic matrices tested, all three viruses were completely inactivated within 180 min at 70 °C.

Enveloped RNA viruses (PEDV, BVDV, PRRSV, SIV, and CDV) were inactivated on dry surfaces at 70 °C, with inactivation times ranging from 90 to 600 min. Moisture, in turn, drastically reduced this time to 5 min at 70 °C. Among organic matrices, BVDV was the most resistant, requiring up to 60 min to be inactivated at 70 °C.

Finally, DNA viruses (BoHV-1 and PPV) showed the greatest overall resistance. Nonetheless, the envelope of BoHV-1 appears to render it more thermolabile than the non-enveloped, structurally robust PPV. For up to 24 h at 70 °C, neither of these two was inactivated on any of the dry surfaces. In contrast, on wet surfaces, BoHV-1 was inactivated within 5 min and PPV within 180 min at 70 °C. Exceptionally, these two viruses were the only ones not inactivated in some organic matrices at 70 °C. Interestingly, across organic matrices, viruses were generally more resistant in feed and straw than in blood. Important exceptions, however, are BoHV-1 and PPV, which were not inactivated in blood until the last time point (180 min) at 70 °C. These differences highlight the importance of directly comparing organic matrices and viruses.

Based on these findings, humid heat treatment at 70 °C for 10 min is highly effective for inactivating enveloped viruses and most non-enveloped viruses, except PPV, making this approach suitable for rapid turnover scenarios. However, if PPV or other non-enveloped DNA viruses are a concern, a more conservative protocol of 70 °C for over 60 min is recommended to increase the likelihood of inactivation. Notably, the protective effect of organic material underscores the critical need for thorough pre-cleaning, as physical removal of debris is essential to maximize the efficacy of any thermal disinfection protocol.

It is essential to note that in this study, we use the term ‘inactivation’ to mean that the infectious virus titer was below the assay’s limit of detection (1.9 log_10_ TCID_50_/mL). Not detected does not imply sterilization or the complete absence of infectious particles. This distinction is crucial for pathogens with a low infectious dose, for which even minimal residual amounts, potentially below our limit of detection, could remain epidemiologically relevant. Accordingly, all operational recommendations should include a conservative safety margin beyond the first non-detected time point.

## Figures and Tables

**Table 1:** Characteristics of viruses (or surrogates of target viruses) employed in the study. Cell lines used for propagation of viruses included Crandell–Rees Feline Kidney (CRFK), Swine Testis (ST), Porcine Kidney (PK-15), African Green Monkey Kidney (VERO 81), Madin–Darby Bovine Kidney (MDBK), Monkey Kidney (MARC-145), and Madin–Darby Canine Kidney (MDCK). Viral stock titers are expressed as the 50% tissue culture infectious dose (TCID_50_) per milliliter.

Viral Family	Target Virus	Surrogate Viruses Employed	Strain	Cell Line Used for Amplification	Virus Titer (TCID_50_/mL)
*Arteriviridae*	Porcine reproductive and respiratory syndrome Virus	Not applied	NVLS	MARC-145	10^4.8^
*Caliciviridae*	Vesicular exanthema of swine virus	Feline calicivirus	FCV2280	CRFK	10^8.2^
*Coronaviridae*	Porcine epidemic diarrhea virus	Not applied	Colorado 2013	VERO 81	10^5.4^
*Flaviviridae*	Classical swine fever virus	Bovine viral diarrhea virus	Singer	MDBK	10^6.2^
*Orthoherpesviridae*	Pseudorabies virus	Bovine alphaherpesvirus 1	Colorado strain	MDBK	10^7.6^
*Orthomyxoviridae*	Swine influenza virus A	Not applied	A/Swine/Iowa/73/H1N1	MDCK	10^6.7^
*Paramyxoviridae*	Nipah virus	Canine distemper virus	Snyder Hill	VERO 81	10^8.1^
*Parvoviridae*	Porcine parvovirus	Not applied	NADL-1 (Mengeling)	ST	10^6.9^
*Picornaviridae*	Foot and mouth disease virus	Senecavirus A1	HI/2012-NADC40	ST	10^8.7^
*Picornaviridae*	Swine vesicular disease virus	Porcine sapelovirus	Smedia (PS27)	PK-15	10^7.7^

**Table 2:** Viral viability of +ssRNA, non-enveloped viruses exposed to temperatures of 70 °C, 60 °C, and 50 °C under dry conditions (up to 1440 min) or under wet and organic conditions (up to 180 min). Average viral titers (log_10_ TCID_50_/mL) are shown for the last time point at which viral detection was observed. Not detected (ND) denotes viral titers below the assay detection threshold (<1.9 log_10_ TCID_50_/mL) at the initial sampling point (5 min).

Virus	Condition	Matrix	Viability Time in Minutes (Viral Titer)			
			70 °C	60 °C	50 °C	~20–22 °C
Feline calicivirus (FCV)	Dry	Plastic	1440 (3.4)	1440 (3.4)	1440 (4.3)	900 (3.8)
		Rubber	1440 (3.3)	1440 (3.3)	1440 (4.3)	1440 (2.8)
		Aluminum	1440 (4.1)	1440 (3.9)	1440 (4.6)	1440 (2.6)
		Stainless steel	1440 (4.3)	1440 (3.5)	1440 (3.8)	1440 (2.6)
	Wet	Plastic	5 (ND)	5 (ND)	15 (3.9)	60 (6.6)
		Rubber	5 (ND)	5 (ND)	15 (4.1)	60 (6.4)
		Aluminum	5 (ND)	5 (ND)	15 (2.8)	60 (6.8)
		Stainless steel	5 (ND)	5 (ND)	15 (3.2)	60 (6.8)
	Organic matter	Starter feed	15 (2.3)	30 (2.7)	180 (1.9)	180 (4.1)
		Finisher feed	30 (2.3)	180 (1.9)	180 (2.8)	180 (5.6)
		Wheat straw	15 (2.8)	30 (2.7)	180 (2.6)	180 (5.8)
		Blood	5 (ND)	15 (1.9)	180 (2.3)	180 (5.8)
Senecavirus A (SVA)	Dry	Plastic	1440 (2.6)	1440 (3.1)	1440 (4.3)	1440 (5.3)
		Rubber	1440 (3.2)	1440 (2.9)	1440 (3.4)	1440 (5.1)
		Aluminum	1440 (3.2)	1440 (3.9)	1440 (4.7)	1440 (4.8)
		Stainless steel	1440 (2.6)	1440 (3.6)	1440 (3.9)	1440 (4.8)
	Wet	Plastic	5 (ND)	5 (ND)	30 (2.3)	60 (6.8)
		Rubber	5 (ND)	5 (ND)	30 (2.5)	60 (6.8)
		Aluminum	5 (ND)	5 (ND)	30 (2.1)	60 (6.8)
		Stainless steel	5 (ND)	5 (ND)	30 (2.1)	60 (6.8)
	Organic matter	Starter feed	30 (4.7)	180 (1.9)	180 (3)	180 (2.9)
		Finisher feed	60 (2.6)	180 (2.6)	180 (3.2)	180 (2.3)
		Wheat straw	60 (2.1)	180 (1.9)	180 (2.1)	180 (1.9)
		Blood	15 (2.1)	60 (2.4)	180 (6.8)	180 (6.8)
Sapelovirus (PSV)	Dry	Plastic	60 (2.2)	120 (2.2)	120 (2.6)	120 (4.8)
		Rubber	90 (2.5)	120 (2.2)	120 (2.9)	120 (4.8)
		Aluminum	120 (2)	120 (2.6)	120 (3.4)	120 (4.8)
		Stainless steel	120 (2)	120 (2.2)	120 (3.4)	120 (4.8)
	Wet	Plastic	5 (ND)	5 (ND)	5 (1.9)	30 (6.1)
		Rubber	5 (ND)	5 (ND)	20 (2.9)	30 (6.2)
		Aluminum	5 (ND)	5 (ND)	5 (ND)	30 (6.5)
		Stainless steel	5 (ND)	5 (ND)	5 (1.9)	30 (6.2)
	Organic matter	Starter feed	15 (3.4)	30 (3.6)	180 (3.3)	180 (4.9)
		Finisher feed	10 (2.2)	60 (3.2)	180 (3)	180 (5.1)
		Wheat straw	10 (2.3)	60 (2.4)	60 (3.1)	180 (5.1)
		Blood	5 (ND)	5 (ND)	15 (3)	180 (4.9)

**Table 3:** Viral viability of +ssRNA, enveloped viruses exposed to temperatures of 70 °C, 60 °C, and 50 °C under dry conditions (up to 1440 min) or under wet and organic conditions (up to 180 min). Average viral titers (log_10_ TCID_50_/mL) are shown for the last time point at which viral detection was observed. Not detected (ND) denotes viral titers below the assay detection threshold (<1.9 log_10_ TCID_50_/mL) at the initial sampling point (5 min).

Virus	Condition	Matrix	Viability Time in Minutes (Viral Titer)			
			70 °C	60 °C	50 °C	~20–22 °C
Porcine epidemic diarrhea virus (PEDV)	Dry	Plastic	150 (2.1)	150 (3.1)	150 (3.1)	180 (2.9)
		Rubber	5 (2.3)	150 (2.1)	150 (2.9)	180 (2.4)
		Aluminum	150 (2.1)	150 (3)	150 (3.3)	180 (2.4)
		Stainless steel	150 (2.2)	150 (3.2)	150 (3.3)	180 (2.4)
	Wet	Plastic	5 (ND)	5 (ND)	10 (1.9)	15 (4.1)
		Rubber	5 (ND)	5 (ND)	10 (2.2)	15 (3.7)
		Aluminum	5 (ND)	5 (ND)	10 (1.9)	15 (3.9)
		Stainless steel	5 (ND)	5 (ND)	10 (1.9)	15 (3.6)
	Organic matter	Starter feed	5 (ND)	5 (ND)	5 (ND)	15 (1.9)
		Finisher feed	5 (ND)	5 (ND)	5 (ND)	15 (1.9)
		Wheat straw	5 (2.1)	5 (2.5)	20 (2.1)	30 (3.3)
		Blood	5 (3.2)	5 (3.1)	60 (3.1)	180 (3.4)
Bovine virus diarrhea virus (BVDV)	Dry	Plastic	210 (2.1)	210 (3.3)	210 (4.2)	240 (1.9)
		Rubber	120 (2)	210 (3.2)	210 (4.3)	180 (2.2)
		Aluminum	210 (2.1)	210 (3.3)	210 (4.2)	240 (1.9)
		Stainless steel	210 (2.1)	210 (3.1)	210 (4.2)	240 (1.9)
	Wet	Plastic	5 (ND)	5 (ND)	15 (2.2)	30 (4.5)
		Rubber	5 (ND)	5 (ND)	20 (1.9)	30 (4.5)
		Aluminum	5 (ND)	5 (ND)	20 (1.2)	30 (4.5)
		Stainless steel	5 (ND)	5 (ND)	20 (1.9)	30 (4.3)
	Organic matter	Starter feed	30 (2.8)	30 (3.2)	30 (3.3)	30 (3.9)
		Finisher feed	30 (2.6)	30 (3.3)	30 (2.8)	30 (3.6)
		Wheat straw	15 (2.8)	15 (3.3)	15 (3.5)	60 (2.6)
		Blood	30 (2.4)	180 (3.8)	180 (3.8)	180 (5.8)
Porcine reproductive and respiratory syndrome virus (PRRSV)	Dry	Plastic	90 (1.9)	90 (2.4)	90 (2.3)	90 (2.1)
		Rubber	90 (1.9)	90 (2.4)	90 (2.2)	90 (2)
		Aluminum	90 (1.9)	90 (2.1)	90 (2.2)	90 (2.1)
		Stainless steel	90 (1.9)	90 (2.2)	90 (2.2)	90 (2.2)
	Wet	Plastic	5 (ND)	5 (ND)	60 (2)	60 (2.6)
		Rubber	5 (ND)	5 (ND)	60 (1.9)	60 (2.3)
		Aluminum	5 (ND)	5 (ND)	60 (1.9)	60 (2.6)
		Stainless steel	5 (ND)	5 (ND)	60 (2)	60 (2.6)
	Organic matter	Starter feed	5 (ND)	5 (ND)	5 (ND)	5 (1.9)
		Finisher feed	5 (ND)	5 (ND)	5 (ND)	5 (ND)
		Wheat straw	5 (ND)	5 (ND)	5 (ND)	5 (ND)
		Blood	5 (ND)	5 (ND)	5 (ND)	5 (ND)

**Table 4:** Viral viability of −ssRNA, enveloped viruses exposed to temperatures of 70 °C, 60 °C, and 50 °C under dry conditions (up to 1440 min) or under wet and organic conditions (up to 180 min). Average viral titers (log_10_ TCID_50_/mL) are shown for the last time point at which viral detection was observed. Not detected (ND) denotes viral titers below the assay detection threshold (<1.9 log_10_ TCID_50_/mL) at the initial sampling point (5 min).

Virus	Condition	Matrix	Viability Time in Minutes (Viral Titer)			
			70 °C	60 °C	50 °C	~20–22 °C
Swine influenza virus A (SIV)	Dry	Plastic	570 (2.1)	540 (3.7)	540 (4.5)	600 (5.4)
		Rubber	570 (2.1)	540 (3.1)	540 (4.5)	600 (5.2)
		Aluminum	570 (1.9)	540 (3.4)	540 (4.4)	600 (5.4)
		Stainless steel	570 (1.9)	540 (3.4)	540 (4.5)	600 (5.2)
	Wet	Plastic	5 (ND)	5 (ND)	60 (3.8)	60 (6.2)
		Rubber	5 (ND)	5 (ND)	60 (3.7)	60 (5.9)
		Aluminum	5 (ND)	5 (ND)	60 (3.8)	60 (6.2)
		Stainless steel	5 (ND)	5 (ND)	60 (3.8)	60 (6.3)
	Organic	Starter feed	5 (3.8)	5 (3.8)	5 (4)	5 (3.8)
		Finisher feed	5 (3.7)	5 (3.6)	5 (3.8)	10 (3.8)
		Wheat straw	5 (3.4)	20 (2.1)	30 (2.6)	180 (4.2)
		Blood	5 (ND)	5 (4.8)	30 (5)	30 (4.7)
Canine distemper virus (CDV)	Dry	Plastic	60 (2.1)	60 (2.5)	60 (2.3)	60 (5.5)
		Rubber	60 (2.1)	60 (2.1)	60 (1.9)	60 (4.7)
		Aluminum	60 (2.4)	60 (2.5)	60 (2.2)	60 (5.6)
		Stainless steel	60 (2.6)	60 (2.1)	60 (1.9)	60 (5.5)
	Wet	Plastic	5 (ND)	5 (ND)	5 (ND)	15 (5.9)
		Rubber	5 (ND)	5 (ND)	5 (ND)	15 (4.7)
		Aluminum	5 (ND)	5 (ND)	5 (ND)	15 (4.2)
		Stainless steel	5 (ND)	5 (ND)	5 (ND)	15 (3.4)
	Organic	Starter feed	5 (ND)	5 (ND)	5 (ND)	60 (3)
		Finisher feed	5 (ND)	5 (ND)	5 (ND)	60 (3)
		Wheat straw	5 (ND)	5 (ND)	5 (ND)	15 (3)
		Blood	5 (ND)	5 (ND)	5 (ND)	5 (2.1)

**Table 5:** Viral viability of dsDNA, enveloped virus exposed to temperatures of 70 °C, 60 °C, and 50 °C under dry conditions (up to 1440 min) or under wet and organic conditions (up to 180 min). Average viral titers (log_10_ TCID_50_/mL) are shown for the last time point at which viral detection was observed. Not detected (ND) denotes viral titers below the assay detection threshold (<1.9 log_10_ TCID_50_/mL) at the initial sampling point (5 min).

Virus	Condition	Matrix	Viability Time in Minutes (Viral Titer)			
			70 °C	60 °C	50 °C	~20–22 °C
Bovine herpesvirus 1 (BoHV-1)	Dry	Plastic	1440 (2.1)	1440 (3.2)	1440 (4.4)	1440 (4.1)
		Rubber	1440 (2.2)	1440 (2.9)	1440 (4.3)	1440 (3.9)
		Aluminum	1440 (1.9)	1440 (3.7)	1440 (4.2)	1440 (4.1)
		Stainless steel	1440 (2.9)	1440 (3.4)	1440 (4.7)	1440 (3.6)
	Wet	Plastic	5 (ND)	5 (2.5)	180 (2.4)	180 (6.7)
		Rubber	5 (ND)	5 (2.6)	180 (1.8)	180 (6.6)
		Aluminum	5 (ND)	5 (2.9)	180 (2.2)	180 (6.8)
		Stainless steel	5 (ND)	5 (2.9)	180 (2.4)	180 (6.8)
	Organic	Starter feed	180 (2.7)	180 (4.2)	180 (5.8)	180 (3.9)
		Finisher feed	180 (2.2)	180 (4.2)	180 (5.8)	180 (2.7)
		Wheat straw	60 (1.9)	180 (4.7)	180 (5.8)	180 (2.7)
		Blood	180 (4.8)	180 (5.8)	180 (5.8)	180 (6.8)

**Table 6:** Viral viability of ssDNA, non-enveloped virus exposed to temperatures of 70 °C, 60 °C, and 50 °C under dry conditions (up to 1440 min) or under wet and organic conditions (up to 180 min). Average viral titers (log_10_ TCID_50_/mL) are shown for the last time point at which viral detection was observed. Not detected (ND) denotes viral titers below the assay detection threshold (<1.9 log_10_ TCID_50_/mL) at the initial sampling point (5 min).

Virus	Condition	Matrix	Viability Time in Minutes (Viral Titer)			
			70 °C	60 °C	50 °C	~20–22 °C
Porcine parvovirus (PPV)	Dry	Plastic	1440 (3.4)	1440 (4.1)	1440 (4)	1440 (4.3)
		Rubber	1440 (3.9)	1440 (4.1)	1440 (4)	1440 (4.2)
		Aluminum	1440 (3.9)	1440 (4.6)	1440 (4.2)	1440 (4.5)
		Stainless steel	1440 (4.2)	1440 (4.4)	1440 (3.9)	1440 (4.4)
	Wet	Plastic	60 (2.9)	60 (3.8)	60 (3.8)	60 (5.9)
		Rubber	60 (3)	60 (3.7)	60 (3.8)	60 (5.8)
		Aluminum	60 (3.1)	60 (3.8)	60 (3.8)	60 (6.1)
		Stainless steel	60 (3.1)	60 (3.8)	60 (3.8)	60 (5.8)
	Organic	Starter feed	5 (ND)	60 (1.9)	180 (2.2)	180 (2.3)
		Finisher feed	30 (2)	180 (2.1)	180 (2.3)	180 (2.2)
		Wheat straw	60 (1.9)	180 (2.6)	180 (2.5)	180 (3.4)
		Blood	180 (2.2)	180 (2.6)	180 (2.2)	180 (4.6)

## Data Availability

The original contributions presented in this study are included in the article.
